# Hepatocellular carcinoma risk in metabolic dysfunction-associated steatotic liver disease with increased alcohol intake (MetALD)

**DOI:** 10.1016/j.jhepr.2025.101639

**Published:** 2025-10-16

**Authors:** Binu V. John, Dustin Bastaich, Elizabeth Paulus, Seth Spector, Bassam Dahman

**Affiliations:** 1 Division of Gastroenterology and Hepatology, Miami VA Medical System, Miami, FL, USA; 2 Division of Digestive Health and Liver Diseases, University of Miami Miller School of Medicine, Miami, FL, USA; 3 Department of Biostatistics, Virginia Commonwealth University, Richmond, VA, USA; 4 Department of Surgery, Miami VA Medical System, Miami, FL, USA; 5 Department of Surgery, University of Miami Miller School of Medicine, Miami, FL, USA; 6 Department of Health Policy, Virginia Commonwealth University, Richmond, VA, USA

**Keywords:** Hepatocellular carcinoma, Alcohol use disorder, Alcohol-associated liver disease, AUDIT-C, Veterans Analysis of Liver Disease, Metabolic and alcohol-associated steatotic liver disease

## Abstract

**Background & Aims:**

Metabolic dysfunction-associated steatotic liver disease (MASLD) with increased alcohol consumption (MetALD) is an important, yet understudied cause of liver disease. This study aimed to examine hepatocellular carcinoma (HCC) rates in patients with MetALD and compare them with MASLD, alcohol-associated liver disease (ALD), and controls.

**Methods:**

We conducted a retrospective cohort study of veterans with MetALD, MASLD, ALD, or no SLD. We used Poisson regression to estimate incidence rates within stratifications of patient characteristics, and a multivariable time-updated Fine and Gray model with death as a competing risk to examine HCC risks.

**Results:**

We included 666,428 participants (392,286 MASLD, 104,065 MetALD, 40,230 ALD, and 129,847 controls) between 1 January 2011 and 31 December 2022, with a follow-up until 31 May 2023. All participants underwent abdominal imaging or transient elastography, confirming the presence (among SLD) or absence (among controls) of steatosis, along with identification of harmful alcohol use and ascertainment of cardiometabolic risk factors. In patients without cirrhosis, the adjusted HCC rates per 100,000 person-years were lowest for MASLD, higher for MetALD, and highest for ALD. The cumulative incidence of HCC at 5 years was highest with ALD, followed by MASLD and MetALD, with rates similar in the latter two groups. At 10 years, the cumulative incidence of HCC per 100,000 individuals was the highest with ALD (1,176.65), followed by MetALD (814.16), and MASLD (762.86).

**Conclusions:**

The risk of HCC associated with MetALD is higher than MASLD, significantly lower than ALD, and likely driven by alcohol. Although HCC rates in non-cirrhotic patients with MetALD are not high enough to justify surveillance, this study identifies a relatively higher risk group compared to MASLD, which warrants counselling on alcohol cessation.

**Impact and implications:**

Limited data exist on the risk of hepatocellular carcinoma (HCC) in patients with MetALD (metabolic dysfunction-associated steatotic liver disease [MASLD] with increased alcohol consumption) and how it compares with other steatotic liver diseases. In this retrospective study of 666,428 patients from the Veterans Analysis of Liver Disease cohort, we observed that the risk of HCC per 100,000 person-years in patients without cirrhosis with MetALD was 80.24 (95% CI 73.23–87.94), which was intermediate between MASLD (71.64, 95% CI 68.14–75.32) and ALD (104.83, 91.59–119.98). The cumulative incidence of HCC per 100,000 persons at 5 years was the highest with ALD (663.79, 581.77–754.83), followed by MASLD (450.71, 428.46–473.89), MetALD (449.89, 407.56-495.74), and controls (76.03, 61.47–93.46). The limitations of the study include its retrospective design and a study population enriched with male veterans with a higher prevalence of metabolic syndrome.

## Highlights

• A study of 666,428 participants (392,286 MASLD, 104,065 MetALD, 40,230 ALD, and 129,847 controls) from the VALID cohort.
• The cumulative incidences of HCC at 5 years per 100,000 individuals were 663.79 for ALD, 450.71 for MASLD, and 449.89 for MetALD.
• At 10 years, the cumulative incidences of HCC per 100,000 individuals were 1,176.65 for ALD, 814.16 for MetALD, and 762.86 for MASLD.
• The higher HCC rate in patients with MetALD compared to MASLD at 10 years is likely explained by cumulative exposure to alcohol.

## Graphical abstract

**Figure:**
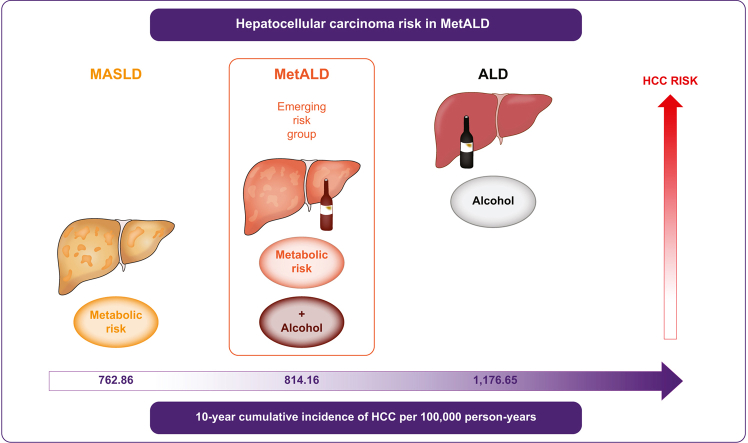

## Introduction

MetALD (metabolic dysfunction-associated steatotic liver disease [MASLD] with increased alcohol consumption), recognized as a dual-etiology steatotic liver disease (SLD) in individuals with steatosis, cardiometabolic risk factors, and alcohol use, is now acknowledged as an important yet understudied contributor to liver disease progression.^1^^,^^2^ The definition of this condition was based on national and international consensus, with the authors acknowledging limited data on its natural history and encouraging the generation of more outcome data.^2^ While the natural history of MASLD and alcohol-associated liver disease (ALD) has been well elucidated, there is limited data on HCC risk in patients with MetALD. In the spectrum of SLD, data from the National Health and Nutrition Examination Survey indicate that MASLD affects 32% of the US population, MetALD affects ∼2.5%, and ALD affects ∼1.2%.^3^ If this is extrapolated to the US population, >5 million patients may have MetALD. Understanding the risk of HCC among patients with MetALD with and without cirrhosis, and how it compares to other steatotic liver diseases, is critical.

Two recent studies from Asia reported a stepwise increase in risk from MASLD to MetALD and then ALD.^4^^,^^5^ However, there are significant differences in clinical presentations, definitions (including BMI cut-off), and outcomes of patients with MASLD (and potentially MetALD) between Asia and the United States. Yun *et al.*^5^ used the Fatty Liver Index as a surrogate for steatosis. Prior published data suggest that blood and anthropometric and laboratory-based risk scores to estimate steatosis demonstrate low correlation with steatosis from imaging, with elevated markers of steatosis observed in many controls with metabolic risk factors and no steatosis.^6^ There are limited data on the prevalence of hepatocellular carcinoma (HCC) in patients with MetALD, including 5- and 10-year estimates, both overall and in important clinical subgroups (males and females, patients with and without cirrhosis, etc.), which are crucial in counselling patients on their risk.

Therefore, the primary aim of this study was to examine the incidence rates of HCC in patients with MetALD with and without cirrhosis. The secondary aim was to compare HCC rates in patients with MetALD with those in patients with MASLD, ALD, and non-SLD controls.

## Patients and methods

### Study design and data sources

We conducted a retrospective cohort study using the Veterans Analysis of Liver Disease (VALID) cohort of well-characterized patients from the Veterans Health Administration (VHA) Corporate Data Warehouse. This cohort included 2.5 million veterans with either an International Classification of Diseases (ICD)-9 or ICD-10 code for chronic liver disease or at least two abnormal alanine aminotransferase (ALT) levels (>40 IU/ml in males and >31 IU/ml in females) 6 months apart. An additional 1.8 million randomly selected individuals with no documented chronic liver disease and normal liver enzymes, but who had an endoscopic examination for any reason, including but not limited to a screening colonoscopy, to document engagement with care in the VHA, were included.[6], [7], [8], [9] Overall, the cohort represented nearly half of the approximately 9 million veterans in the VHA.

This study period included participants from 1 January 2011 to 31 December 2022, with a follow-up period of 31 May 2023, which was different from the prior publication validating the cohort that included patients from 2011.^6^ The number of patients excluded for each reason also varied between publications based on the sequence in which the exclusions were applied. The institutional review boards of the participating Veterans Affairs Medical Center approved the study and waived the requirement for informed consent.

### Subject identification, inclusion, and exclusion criteria

Eligibility criteria included patients aged ≥18 years with VALID who underwent abdominal imaging (for any indication) or transient elastography during the study period.^10^

Patients who did not undergo imaging or elastography were excluded because their steatosis status was not known. We identified patients with SLD (MASLD, MetALD, or ALD) using natural language processing, which was able to identify steatosis from abdominal imaging reports with a high degree of accuracy.^6^ Steatosis was also identified based on a controlled attenuation parameter (CAP) score of >288 dB/s from elastography reports.^6^^,^^10^ We combined the identification of steatosis with documented cardiometabolic risk factors (CMRFs) to identify patients with MASLD. We then used the Alcohol Use Disorders Identification Test-Concise (AUDIT-C) scores to identify patients with harmful alcohol use.^11^ AUDIT-C is a brief evidence-based screening test to identify patients with unhealthy alcohol use that is obtained at least annually for all patients engaged with care in the VHA. It correlates well with the amount of alcohol use and is recommended by the US Preventive Task Force to be superior to self-reported alcohol use. A positive AUDIT-C score was defined as ≥4 for males and ≥3 for females.[11], [12], [13] Patients with steatosis and CMRF with low AUDIT-C were determined to have MASLD; and those with steatosis, CMRF, and positive AUDIT-C were determined to have MetALD. ALD was defined as participants without CMRF and elevated AUDIT-C scores ≥4 in males and ≥3 in females in the 5 years preceding the identification of steatosis.^2^ Additionally, based on the revised guidelines recommending that patients with severe alcohol use be classified as ALD regardless of CMRFs, we defined elevated AUDIT-C scores ≥8 in males and ≥6 in females as having an alcohol use disorder.^6^ The control group was defined as participants who underwent abdominal imaging with no evidence of hepatic steatosis on imaging, did not have an elevated CAP score (>288 dB/s), and had no elevation in ALT before or at baseline. The above algorithm combining natural language processing with CMRF and AUDIT-C identified patients with MASLD, MetALD, ALD, and no SLD with a high degree of accuracy compared to blinded chart reviews and detected >10 times more patients with SLD than those identified using ICD-9/-10 codes.^6^

Baseline was defined as the date of the first documentation of steatosis, and for controls, the date of imaging documenting the absence of steatosis.

### Covariates, exposures, and outcomes

Patient demographics (age, sex, and self-reported race/ethnicity) and baseline measurements (BMI, diabetes mellitus, cirrhosis, smoking status, and laboratory results) were extracted closest to the baseline date (within 1 year) for all participants.[14], [15], [16] The outcome of interest was HCC, which was identified using an ICD-9 code of 155.0 or ICD-10 code of C22.0, which has a high positive predictive value in the VHA.^17^^,^^18^ Participants were followed up until death or at the end of the study (31 May 2023).

### Ethical compliance

All procedures performed in this study involving human participants were in accordance with the ethical standards of the institutional and/or national research committee and the 1964 Helsinki Declaration and its later amendments or comparable ethical standards.

### Statistical analysis

Descriptive statistics of the baseline characteristics were compared between the three SLD groups and the controls in the full sample. ANOVA was used to compare continuous variables, whereas the Χ^2^ test was used for binary and categorical variables.

We calculated the propensity scores for each etiology and applied inverse probability of treatment weighting (IPTW) to create a pseudo-sample balanced for baseline covariates.

HCC incidence rates were estimated and compared across the three etiologies of steatosis: MASLD, MetALD, and ALD. Incidence rates were reported as events per 100,000 person-years (PY). Poisson regression models were used to estimate the incidence rates and 95% CIs within patient characteristic stratifications.

To account for potential heterogeneity, we conducted stratified analyses by cirrhosis status to determine whether the risk of HCC differed between patients with and without cirrhosis. Given that cirrhosis is the strongest known predictor of HCC, this stratification would help clarify whether the observed risk differences and trends between MASLD to MetALD and ALD persisted independently of cirrhosis status.

Additionally, subgroup analyses according to patient demographics (age, sex, race/ethnicity, and diabetes status) were performed to identify whether certain populations were disproportionately affected by the MetALD-related HCC risk. By examining variations in risk across these strata, we aimed to assess whether the stepwise increase in HCC risk from MASLD to MetALD to ALD was consistent across diverse patient groups.

Using a multivariable time-updated Fine and Gray competing risk model with death as a competing risk, we compared the hazard of HCC according to etiology. The model was further adjusted for sex, race, age, cirrhosis, diabetes, BMI, tobacco use, ALT, and aspartate aminotransferase (AST) levels, and platelet count. These covariates were updated at the time of cirrhosis in participants who developed cirrhosis after baseline. We also visualized the differences in HCC by etiology using a plot of the cumulative incidence of HCC. Pairwise differences by etiology were compared using the Bonferroni adjustment for multiple comparisons.

We also performed the following sensitivity analyses: First, to account for potential patients with cirrhosis that may not be identified using ICD-9/-10 codes, we performed a sensitivity analysis by expanding the definition of cirrhosis, including patients with either ICD-9/-10 codes for cirrhosis, an elevated Fib-4 level >2.67, or a platelet count <150 × 10^9^/ml.

Second, to categorize only patients with sustained harmful alcohol use as MetALD or ALD, we defined harmful alcohol use as ≥2 years of elevated AUDIT-C in the sensitivity analysis. Third, as not all patients in the original cohort underwent testing for viral hepatitis B and C, we performed a subgroup analysis including only patients who tested negative for hepatitis B and C.

Statistical significance was defined as *p* <0.05. Statistical analyses were performed using SAS (version 9.4; SAS Inc., Cary, NC, USA).

## Results

### Study population and baseline characteristics

**Fig. 1:**
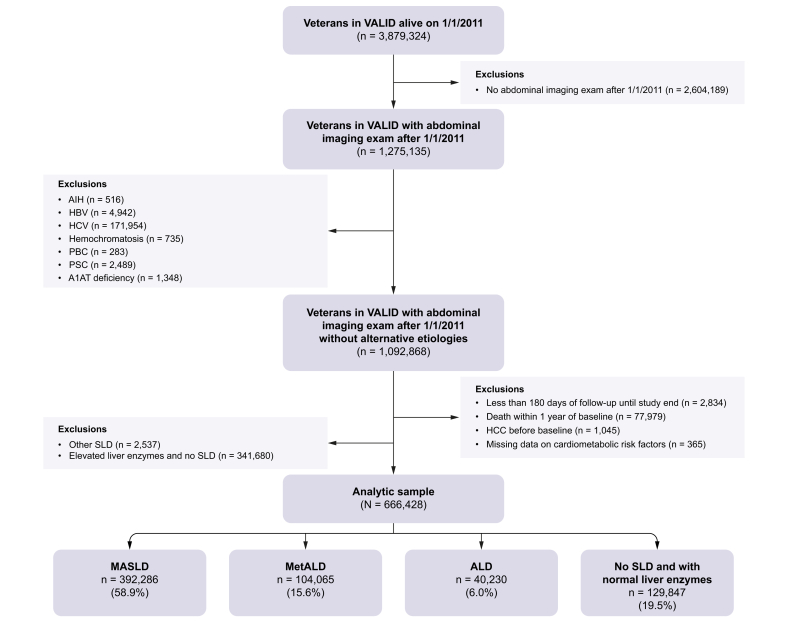
Study flow diagram.

Of the 3,879,324 veterans in VALID who were alive on 1 January 2011, 3,395,823 were engaged in VA care after the start date of the study (Fig. 1). From this group, we identified participants who had undergone an abdominal imaging examination since 1 January 2011 (n = 1,275,135). After excluding participants with alternative etiologies including autoimmune hepatitis, hepatitis B/C, hemochromatosis, primary biliary cholangitis, and primary sclerosing cholangitis, there remained 1,092,868 Veterans. We excluded patients with <180 days of follow-up until the end of the study (n = 2,834), those who died within 1 year of documented steatosis (n = 77,979), those with HCC before documented steatosis (n = 1,045), and those with missing CMRF data (n = 365). We also excluded participants with SLD other than MASLD, MetALD, or ALD, including those with steatosis but without CMRFs or harmful alcohol use (n = 2,537), and those with elevated liver enzymes but without steatosis (n = 341,680). After exclusion, there were 666,428 participants in our analysis: 392,286 with MASLD, 104,065 with MetALD, 40,230 with ALD, and 129,847 controls with no steatosis and normal liver enzymes.

Patients with ALD were younger, with a mean age of 55.4 years (SD 12.1), than those aged >60 years in the other groups (Table 1). The three SLD groups had >90% males, consistent with the veteran population; however, the non-SLD control group had a relatively lower prevalence of males (80.6%). Cirrhosis at baseline was most likely in the ALD group (11.7%) and the least likely in the non-SLD group (0.7%). Diabetes was most prevalent in the MASLD group (24.1%), followed by the MetALD (13.8%), ALD (10.0%), and non-SLD (10.4%) groups. Transaminases were highest in the ALD group, followed by the MetALD and MASLD groups, and lowest in the non-SLD group.

**Table 1:** Baseline descriptive statistics.

Variables	MASLD	MetALD	ALD	Controls	*p* value
	(n = 392,286)	(n = 104,065)	(n = 40,230)	(n = 129,847)	
Age (years), mean (SD)	60.8 (13.1)	58.9 (13.2)	55.4 (12.1)	62.2 (14.8)	<0.0001
Sex, N (%)					<0.0001
Male	357,457 (91.1)	96,963 (93.2)	37,680 (93.7)	104,619 (80.6)	
Female	34,829 (8.9)	7,102 (6.8)	2,550 (6.3)	25,228 (19.4)	
Race/ethnicity, n (%)					<0.0001
White	243,884 (62.2)	65,830 (63.3)	24,699 (61.4)	73,004 (56.2)	
Black	60,517 (15.4)	16,311 (15.7)	6,626 (16.5)	31,126 (24.0)	
Hispanic/Latino	32,823 (8.4)	7,756 (7.5)	3,149 (7.8)	7,229 (5.6)	
Other/missing	55,062 (14.0)	14,168 (13.6)	5,756 (14.3)	18,488 (14.2)	
BMI, n (%)					
Underweight	1,750 (0.5)	834 (0.8)	752 (1.9)	2,077 (1.6)	<0.0001
Normal	36,567 (9.3)	14,029 (13.5)	9,347 (23.2)	34,266 (26.4)	
Overweight	105,712 (27.0)	32,300 (31.0)	14,486 (36.0)	54,088 (41.7)	
Obese	248,257 (63.3)	56,902 (54.7)	15,645 (38.9)	39,416 (30.4)	
Steatosis classification method					—
Imaging only	380,774 (97.1)	100,746 (96.8)	39,120 (97.2)	0	
CAP Score (>288 dBs) only	1,046 (0.3)	305 (0.3)	102 (0.3)	0	
Both	10,466 (2.7)	3,014 (2.9)	1,008 (2.5)	0	
Neither	0	0	0	129,847 (100)	
Cirrhosis at baseline, n (%)	14,196 (3.6)	4,733 (4.6)	4,722 (11.7)	930 (0.7)	<0.0001
Tobacco use, n (%)	281,453 (71.8)	82,513 (79.3)	34,117 (84.8)	88,199 (67.9)	<0.0001
Diabetes, n (%)	94,377 (24.1)	14,328 (13.8)	4,023 (10.0)	13,521 (10.4)	<0.0001
Platelet count, n (%)					
<50 k	599 (0.2)	191 (0.2)	231 (0.6)	95 (0.1)	
50–150 k	32,758 (8.4)	9,915 (9.5)	6,173 (15.3)	6,983 (5.4)	<0.0001
≥150 k	358,929 (91.5)	93,959 (90.3)	33,826 (84.1)	122,769 (94.6)	
Baseline lab results, mean (SD)					
Alanine aminotransferase (IU/ml)	37.9 (30.5)	42.5 (34.6)	49.3 (45.8)	19.4 (10.4)	<0.0001
Aspartate aminotransferase (IU/ml)	29.7 (24.0)	35.5 (32.4)	50.8 (56.2)	20.8 (10.1)	<0.0001
Platelet count	212.3 (47.4)	209.7 (48.3)	201.0 (54.1)	217.8 (45.4)	<0.0001

Note: *p* values for continuous measurements were computed using ANOVAs, and Χ^2^ tests were used for categorical measurements. Tobacco use indicates current or former users. ALD, alcohol-associated liver disease; MASLD, metabolic dysfunction-associated steatotic liver disease; MetALD, metabolic dysfunction and alcohol-associated steatotic liver disease.

### HCC incidence rates of MASLD, MetALD, and ALD

**Fig. 2:**
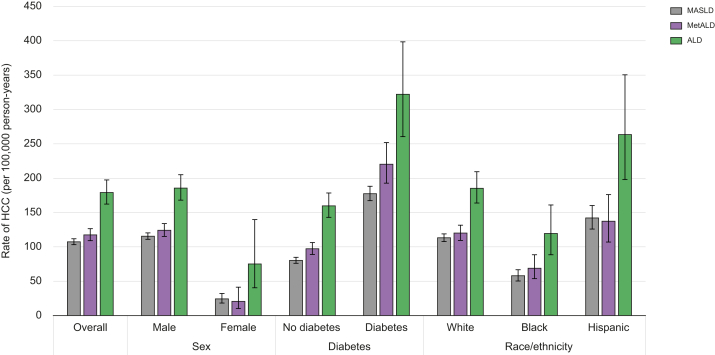
Stratified Incidence rates of HCC per 1,000 person-years among patients with MASLD, MetALD, and ALD. Median Incidence rate of HCC with bars representing 95% confidence intervals estimated by Poisson regression models.ALD, alcohol-associated liver disease; HCC, hepatocellular carcinoma; MASLD, metabolic dysfunction-associated steatotic liver disease; MetALD, metabolic dysfunction and alcohol-associated steatotic liver disease.

The overall HCC incidence rates per 100,000 PY in the study population increased from MASLD (107.44, 95% CI 103.22–111.84, reference) to MetALD (117.48, 95% CI 109.48–126.52, *p* = 0.04) and ALD (179.01, 95% CI 162.26–197.48, *p* <0.0001; Table 2 and Fig. 2). In the subgroup of patients without baseline cirrhosis, these rates were lowest per 100,000 PY for MASLD (71.64, 95% CI 68.14–75.32, reference), intermediate for MetALD (80.24, 95% CI 73.23-87.94, *p* = 0.03), and highest for ALD (104.83, 95% CI 91.59–119.98, *p* <0.0001). Unsurprisingly, the rate of HCC was ∼10-fold higher in patients with cirrhosis. However, HCC rates per 100,000 PY among patients with cirrhosis were highest in MetALD and MASLD, followed by ALD.

**Table 2:** Incidence rate of HCC (per 100,000 person-years) by etiology.

Etiology	MASLD		MetALD		ALD	
	IR (95% CI) (per 100,000 PY)	*p* value	IR (95% CI) (per 100,000 PY)	*p* value	IR (95% CI) (per 100,000 PY)	*p* value
Overall	107.44 (103.22, 111.84)	REF	117.48 (109.08, 126.52)	0.0380	179.01 (162.26, 197.48)	<0.0001
Age (year)						
≤45	17.50 (13.50, 22.68)	REF	12.54 (7.28, 21.60)	0.2788	56.91 (39.03, 82.99)	<0.0001
46–64	87.50 (81.94, 93.44)	REF	102.87 (91.62, 115.50)	<0.0001	187.50 (164.94, 213.15)	<0.0001
≥65	163.79 (155.54, 172.47)	REF	187.75 (170.19, 207.13)	0.0159	272.55 (230.60, 322.13)	<0.0001
Sex						
Male	115.58 (110.99, 120.36)	REF	124.22 (115.29, 133.84)	0.0961	185.62 (168.04, 205.02)	<0.0001
Female	24.23 (18.26, 32.15)	REF	20.67 (10.34, 41.34)	0.6779	75.16 (40.45, 139.68)	0.0011
Cirrhosis baseline						
No Cirrhosis	71.64 (68.14, 75.32)	REF	80.24 (73.23, 87.94)	0.0331	104.83 (91.59, 119.98)	<0.0001
Cirrhosis	1,061.91 (992.76, 1,135.88)	REF	1,078.13 (950.05, 1,223.48)	0.8358	887.92 (769.66, 1,024.34)	0.0264
Race/ethnicity						
White	113.22 (107.77, 118.94)	REF	120.04 (109.49, 131.61)	0.2718	185.24 (163.85, 209.43)	<0.0001
Black	57.96 (50.31, 66.77)	REF	69.02 (53.81, 88.52)	0.2319	119.41 (88.55, 161.03)	<0.0001
Hispanic	142.08 (125.98, 160.23)	REF	137.33 (107.09, 176.11)	0.8094	263.46 (198.06, 350.44)	<0.0001
Other/missing	113.39 (102.13, 125.90)	REF	148.17 (123.90, 177.20)	0.0114	171.90 (131.37, 224.93)	0.0047
BMI						
Obese	103.50 (98.34, 108.92)	REF	107.05 (96.37, 118.91)	0.5715	179.65 (153.74, 209.93)	<0.0001
Overweight	111.85 (103.72, 120.62)	REF	125.93 (110.88, 143.04)	0.1164	190.73 (162.60, 223.72)	<0.00001
Normal	123.84 (109.00, 140.71)	REF	138.26 (114.31, 167.23)	0.3462	152.96 (122.69, 190.69)	0.1043
Underweight	87.14 (41.52, 182.89)	REF	166.10 (79.24, 348.16)	0.2275	279.94 (150.73, 519.93)	0.0179
Diabetes						
No diabetes	80.36 (76.09, 84.87)	REF	97.27 (88.97, 106.34)	0.0003	159.73 (142.99, 178.44)	<0.0001
Diabetes	177.45 (167.27, 188.26)	REF	220.30 (192.73, 251.82)	0.0037	322.15 (260.47, 398.43)	<0.0001

Unadjusted incidence rates and confidence intervals were computed using Poisson regression. ALD, alcohol-associated liver disease; HCC, hepatocellular carcinoma; IR, incidence rate; MASLD, metabolic dysfunction-associated steatotic liver disease; MetALD, metabolic dysfunction and alcohol-associated steatotic liver disease; PY, person-years.

The increasing trend in HCC rates from MASLD to MetALD to ALD was consistent across most age, sex, race/ethnicity, and diabetes subgroups of the cohort (Table 2 and Fig. 2). For example, the rates of HCC per 100,000 PY among non-Hispanic White individuals were lower in MASLD (113.22, 95% CI 107.77–118.94) and MetALD (120.04, 95% CI 109.49–131.61) than in ALD (185.24, 95% CI 163.85–209.49). Similarly, among non-Hispanic Black individuals, the rates of HCC per 100,000 PY were lower than those in Whites, and increased from MASLD (57.96, 95% CI 50.31–66.77) and MetALD (69.02, 95% CI 53.81–88.52) to ALD (119.41, 95% CI 88.55–161.03). HCC rates among all three groups were highest among Hispanic individuals, with rates per 100,000 PY increasing from MetALD (137.33, 95% CI 107.09–176.11) and MASLD (142.08, 95% CI 125.98–160.23) and highest with ALD (263.46, 95% CI 198.06–350.44).

The HCC rates were higher in patients with diabetes than in those without; however, increasing HCC rates from MASLD to MetALD to ALD were observed in patients with and without type 2 diabetes. Among patients without diabetes, HCC rates per 100,000 PY were the lowest in MASLD, intermediate in MetALD, and highest in ALD. A significant increase was observed across all three steatotic liver diseases, including MetALD, with concomitant diabetes (Fig. 2). Even among patients with diabetes, rates per 100,000 PY increased from MASLD to MetALD and were the highest with ALD. Most patients with HCC were diagnosed using imaging, with only 215 patients (6%) requiring a biopsy for diagnosis.

### Cumulative HCC rates of MASLD, MetALD, and ALD at 5 and 10 years

**Fig. 3:**
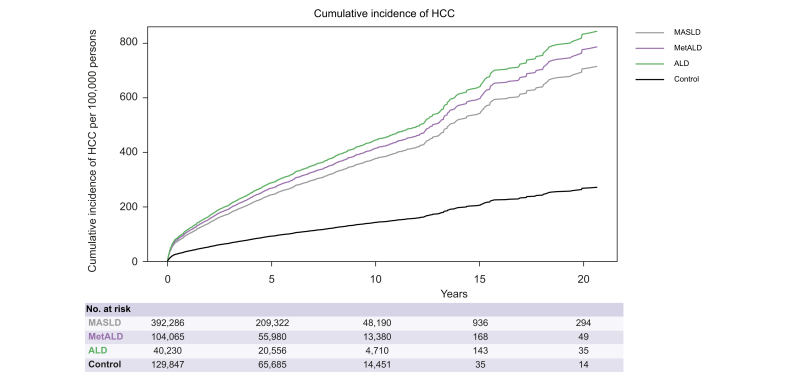
Cumulative incidence of HCC per 100,000 person-years by steatosis etiology among patients with MASLD, MetALD, and ALD using multivariable time-updated Fine and Gray competing risk model with death as a competing risk. ALD, alcohol-associated liver disease; HCC, hepatocellular carcinoma; MASLD, metabolic dysfunction-associated steatotic liver disease; MetALD, metabolic dysfunction and alcohol-associated steatotic liver disease.

The cumulative rates of HCC at 5 and 10 years mirrored the trends in annual incidence rates. The cumulative rates of HCC (per 100,000 persons) at 5 years were similar for MASLD (450.71,95% CI 428.46–473.89) and MetALD (449.89, 95% CI 407.56–495.74), and highest for ALD (663.79, 95% CI 581.77–754.83), compared with controls (Table S1 and Fig. 3). The cumulative rates of HCC at 10 years increased steeply from MASLD to MetALD, while it remained the highest for patients with ALD. We examined the annual rates of imaging, including abdominal ultrasound, CT, and MRI, to identify differences in the frequency of imaging between the groups. We observed that imaging rates were the highest among patients with ALD, but those with MASLD and MetALD underwent similar rates of imaging throughout the study period (Table S2).

### HCC risk in patients with MetALD compared with MASLD, ALD, and non-SLD groups

Compared with the non-SLD groups (reference), the hazard of HCC increased 2.9-fold in patients with MetALD (adjusted hazard ratio [aHR] 2.90, 95% CI 2.71–3.10, *p* <0.0001). The HCC risk among MetALD was intermediate between that of MASLD (aHR *vs.* controls 2.64, 95% CI 2.46–2.82, *p* <0.0001) and ALD (aHR 3.11, 95% CI 2.91–3.33, *p* <0.0001) after adjusting for age, sex, race, cirrhosis, diabetes, BMI, tobacco use, ALT, AST, and platelet count (Table 3).

**Table 3:** Adjusted hazard of HCC from multivariable time-updated Fine and Grey competing risk model with death as a competing risk with IPTW.

Variable	aHR (95% CI)	*p* value
Total participants	666,428	
Number of events (HCC)	3,584	
Etiology (Ref = control)		
MASLD	2.64 (2.46, 2.82)	<0.0001
MetALD	2.90 (2.71, 3.10)	<0.0001
ALD	3.11 (2.91, 3.33)	<0.0001
Age (per 10 years)	1.48 (1.46, 1.51)	<0.0001
Sex (male *vs.* female)	2.16 (1.93, 2.41)	<0.0001
Race/ethnicity (Ref = white)		
Black	0.79 (0.75, 0.84)	<0.0001
Hispanic	1.30 (1.23, 1.38)	<0.0001
Other/missing	1.06 (1.01, 1.11)	0.0169
Cirrhosis	13.77 (13.26, 14.29)	<0.0001
Diabetes	1.48 (1.42, 1.53)	<0.0001
BMI (Ref = normal)		
Obese	1.23 (1.17, 1.30)	<0.0001
Overweight	1.18 (1.13, 1.24)	<0.0001
Underweight	1.14 (0.98, 1.32)	0.0976
Tobacco use	1.03 (0.99, 1.07)	0.2083
ALT	1.002 (1.001, 1.002)	<0.0001
AST	1.001 (1.001, 1.002)	<0.0001
Platelet count (Ref ≥150 k)		
<50 k	2.61 (2.32, 2.93)	<0.0001
50–150 k	1.67 (1.60, 1.73)	<0.0001

The IPTW was weighted for age, sex, race/ethnicity, and cirrhosis. Age, cirrhosis, diabetes, BMI, tobacco use, ALT and AST levels, and platelet counts were updated at the time of cirrhosis in participants who developed cirrhosis at least 90 days after their baseline abdominal imaging examination. aHR, adjusted hazard ratio; ALD, alcohol-associated liver disease; ALT, alanine aminotransferase; AST, aspartate aminotransferase; HCC, hepatocellular carcinoma; IPTW, inverse probability treatment weight; MASLD, metabolic dysfunction-associated steatotic liver disease; MetALD, metabolic dysfunction and alcohol-associated steatotic liver disease.

Pairwise comparisons showed statistically significant differences between the three SLD groups using a Bonferroni-adjusted *p* value of 0.0083. Patients with MetALD had a higher risk of HCC than those with MASLD (aHR 1.10, 95% CI 1.05–1.15, *p* <0.0001), whereas the HCC risk associated with ALD was higher than that of both MetALD (aHR 1.07, 95% CI 1.03–1.12, *p* = 0.001) and MASLD (aHR 1.18, 95% CI 1.13–1.24, *p* <0.0001, Table S3).

### Sensitivity and subgroup analysis

In our first sensitivity analysis, we expanded the definition of cirrhosis to include those with either ICD-9/-10 codes for cirrhosis, an elevated Fib-4 >2.67, or a platelet count <150 × 10^9^/ml (Table S4). We observed higher hazard ratios of all three SLD groups but continued to observe that the HRs retained the relationship with HCC, as in the original analysis, with ALD being the highest, followed by MetALD and then MASLD. We observed a lower HR for cirrhosis than in the original analysis.

This change in cirrhosis definition led to a lower HR for cirrhosis (aHR 6.29, 95% CI 6.05–6.54) compared with the original analysis.

In our second sensitivity analysis, we defined ALD and MetALD as those requiring two or more elevated AUDIT-C values. This resulted in the following change in classification: previously defined ALD had 13,967 moves to MASLD, 13,908 moved to MetALD, and 810 moved to other SLD, while previously defined MetALD had 63,183 moves to MASLD (Table S5). The HR of etiologies remained similar to the primary analysis, and the relationship between increasing HCC rates and controls from MASLD (aHR 2.75, 95% CI 2.57–2.94), MetALD (aHR 3.06, 95% CI 2.86–3.27), and ALD (aHR 3.14, 95% CI 2.93–3.36) remained. MetALD and ALD had significantly higher HCC rates than MASLD, but there was no significant difference between MetALD and ALD in this sensitivity analysis.

We performed a subgroup analysis that included only patients in the original cohort who underwent testing for viral hepatitis B and C. A total of 74,986 patients were tested for HBV (46,426 MASLD, 13,345 MetALD, 6,562 ALD, and 8,653 controls) and 31,034 were tested for HCV (18,223 MASLD, 4,813 MetALD, 2,670 ALD, and 5,328 controls). There were 9,540 tested for both, breaking down into 5,713 MASLD, 1,648 MetALD, 1,002 ALD, and 1,177 controls. Table S6 shows the results of the Cox model for the subsets tested for HBV and HCV. MASLD was used as the reference group because there were no HCC events in the 1,177 controls. MetALD tended to have a higher HCC rate than MASLD, but this was not statistically significant; however, the sample was likely underpowered. However, ALD had a significantly higher rate of HCC than MASLD. Finally, we performed a subgroup analysis of the female participants. The HCC rates for all three groups were lower in females than in males. Similar trends were observed, with HCC rates being highest in patients with ALD. However, HCC rates among females were slightly higher in MASLD than MetALD, but was not statistically significant (24.23 *vs.* 20.67 per 100,000 PY, *p* = 0.68). Similar trends were observed at both 5 years (ALD 304.15, MASLD 84.98, and MetALD 79.08 per 100,000 PY) and 10 years (ALD 893.55, MASLD 201.46, and MetALD 179.46 per 100,000 PY, Table S7).

## Discussion

In this national cohort of veterans, we found that patients with MetALD had a slightly higher rate of HCC than those with MASLD but significantly lower rates than those with ALD. This increase from MASLD to MetALD to ALD was observed among both males and females, White and Black individuals, and among those with and without diabetes, as well as among patients without cirrhosis. The higher risk of HCC in MetALD than in MASLD is consistent with the additional carcinogenic potential of alcohol.^19^ Alcohol is an independent risk factor for the development of HCC, with a relative risk of over two-fold for heavy drinkers compared with non-drinkers. The relative risk also slightly increased in occasional drinkers.^20^^,^^21^ When examining HCC at 5 and 10 years, the rates of HCC in MetALD were similar to those in MASLD at 5 years, but higher at 10 years. This may be due to cumulative exposure to alcohol use, whereby the increased risk of HCC may take >5 years to manifest. We examined the annual rates of imaging, including abdominal ultrasound, CT, and MRI, in the three groups. We observed that imaging rates were highest among patients with ALD, but those with MASLD and MetALD underwent similar rates of imaging throughout the study period, suggesting that the higher rates of HCC in MetALD than in MASLD at 10 years, but not at 5 years, is not attributable to more frequent imaging.

Interestingly, among patients with cirrhosis, ALD had lower rates than MASLD or MetALD. This may be because once cirrhosis is established, the HCC risk may be more likely to be driven by fibrosis than by ongoing alcohol use. It is also possible that patients with ALD may have reduced alcohol use after the diagnosis of HCC, while there are fewer effective interventions to improve metabolic syndrome in MASLD and MetALD patients. The lower HCC rates in ALD cirrhosis compared with MASLD cirrhosis have been described in previous publications.^22^ Our data corroborate known risk factors for HCC identified in prior studies, including advanced age, male sex, type 2 diabetes, and inverse association with platelet counts.^7^^,^^17^^,^^18^^,^^22^

MASLD and ALD share a similar clinical course, with progression from steatosis to compensated cirrhosis, hepatic decompensation, and the development of HCC. Several studies have shown that the risk factors of both conditions frequently co-exist and have a synergistic effect in driving liver disease development and progression.[23], [24], [25] For example, patients with alcohol-associated liver disease may have hyperlipidemia and diabetes mellitus that is driven by alcohol. However, MetALD is a diagnosis based on expert opinion, with limited data at its introduction to justify its establishment as a separate entity. The data presented here suggest that MetALD has a higher HCC risk than MASLD, and a lower risk than ALD, suggesting that increasing the amount of alcohol progressively increases the risk of HCC.

We believe that the quantification of the incidence rates of HCC in MetALD is novel. Our findings are consistent with those of two Asian studies that reported a stepwise increase in risk from MASLD to MetALD and then ALD.^4^^,^^5^ The methods presented here offer advantages over these prior efforts. We included only patients documented to have steatosis on imaging in the SLD group, and those with a documented absence of steatosis as controls. Yun *et al.*^5^ used the Fatty Liver Index as a surrogate for steatosis. Anthropometric and laboratory-based risk scores to estimate steatosis demonstrated low correlation with steatosis from imaging, with elevated markers of steatosis observed in many controls with metabolic risk factors and no steatosis.^6^ Furthermore, there were significant differences in clinical presentations, definitions (including BMI cut-off), and outcomes of patients with MASLD (and potentially MetALD) in Asia and the USA.

### Limitations

Because of its retrospective nature, the study may have been affected by residual confounding as a result of unobserved differences between the participants. We used inverse probability of treatment weighting to minimize the differences between the groups. Because of the low incidence of HCC in this predominantly non-cirrhotic cohort, a prospective study enrolling several hundred thousand participants would be challenging. Second, we assessed alcohol use using AUDIT-C, which was not included in the Delphi consensus. Quantifying alcohol intake in clinical practice remains challenging owing to year-to-year variations, underreporting, and recall bias. Checking phosphatidylethanolamine (peth) levels in patients with MetALD has been recommended as a method to corroborate alcohol use. In fact, AUDIT-C has been shown to have a superior correlation with peth levels compared with self-reported alcohol consumption.[11], [12], [13]^,^^26^ However, peth levels were unavailable for this retrospective study. Third, although the veteran cohort was predominantly male, the large sample size ensured that >65,000 women were included in the study. Our subgroup analysis among females indicated that patients with ALD had the highest rates of HCC, followed by MASLD and MetALD. However, veterans have a higher prevalence of metabolic syndrome and alcohol consumption than the general US population.^27^^,^^28^ Therefore, the incidence of HCC in this cohort may be higher than that in the general population. Fourth, the patients did not undergo HCC surveillance, and some patients with HCC may have been missed without routine surveillance. However, HCC surveillance is not recommended in non-cirrhotic SLD, and if these patients are diagnosed with HCC, it would likely occur during routine clinical care. We examined the annual rates of imaging and found that patients with ALD had slightly higher rates of imaging but identified no differences between imaging rates among patients with MASLD and MetALD. We believe that the slightly higher imaging rates in the ALD group will not fully account for the significantly higher HCC rates. Finally, our definition of cirrhosis in the primary analysis was captured using ICD-9/-10 codes, which may have missed some patients. To account for this, we performed a sensitivity analysis, including patients with ICD-9/-10 codes, Fib-4 >2.67, or platelet count <150 × 10^9^ to identify cirrhosis (Table S3), which corroborated the findings observed in the primary analysis.

Our study has several strengths. Although the HCC incidence rates were low among patients without cirrhosis with MetALD, the large sample size helped obtain estimates. The VHA routinely documents alcohol use at least annually for all veterans accessing care, minimizing bias. We defined SLD as patients with steatosis on abdominal imaging that was identified with a high degree of accuracy from imaging reports using natural language processing. The ability to search for all abdominal imaging reports, regardless of whether the studies were performed for liver-related indications, helps improve generalizability. We identified steatosis using a CAP score of 288 dB/s, as recommended by the American Association for the Study of AASLD guidelines. The majority of participants with steatosis were identified using imaging only, or imaging and CAP scores. Only 1,453 patients were observed to have steatosis with CAP alone. Examining steatosis using a lower cut-off of 242 dB/s only changed the classification of 101 patients from the control group to the steatosis group, indicating that the cut-off used for CAP scores had minimal effects on the analysis performed in this study.

Additionally, because we had access to the complete medical records of the participants, we were able to determine the presence or absence of cardiometabolic risk factors in >99% of the potential study participants.

## Conclusions

HCC rates in patients with MetALD were higher than those in patients with MASLD but lower than those in patients with ALD. Our data suggest that, although HCC rates in patients without cirrhosis with MetALD are not high enough to justify surveillance, this identifies a relatively higher risk group compared to MASLD, which warrants counselling on alcohol cessation.

## Abbreviations

aHR, adjusted hazard ratio; ALD, alcohol-associated liver disease; ALT, alanine aminotransferase; AST, aspartate aminotransferase; AUDIT-C, Alcohol Use Disorders Identification Test-Concise; CAP, controlled attenuation parameter; CMRFs, cardiometabolic risk factors; HCC, hepatocellular carcinoma; ICD, International Classification of Diseases; IPTW, inverse probability treatment weight; MASLD, metabolic dysfunction-associated steatotic liver disease; MetALD, metabolic dysfunction and alcohol-associated steatotic liver disease; peth, phosphatidylethanolamine; PY, person-years; SLD, steatotic liver disease; VALID, Veterans Analysis of Liver Disease; VHA, Veterans Health Administration.

## Financial support

This study was supported by the VA Office of Research and Development CSR and D VA MERIT I01CX002654 01A1 (BVJ). Services supporting this analysis and the interpretation of data for this research project were also generated by the South Florida VA Foundation for Research and Education and the VCU Massey Cancer Center Biostatistics Shared Resource, which were supported in part by funding from the NIH-NCI Cancer Center Support Grant P30 CA016059 (Dahman). The funding agencies played no role in any aspect of the study, including the design, conduct, management, analysis, reporting, or decision to submit the manuscript for publication.

## Authors’ contributions

Had full access to all the data in the study and were responsible for the integrity of the data and the accuracy of the data analysis BVJ, BD. Concept and design: BVJ. Acquisition, analysis, and interpretation of data: BVJ, DB, BD. Drafting of the manuscript: BVJ. Critical revision of the manuscript for important intellectual content: all authors. Statistical analysis: DB, BVJ, BD. Obtained funding: BVJ, BD. Administrative, technical, or material support: all authors. Supervision: BVJ.

## Data availability

The US Department of Veterans Affairs (VA) places legal restrictions on access to veterans’ healthcare data, including identifying and sensitive patient information. The analytic datasets used in this study were not permitted to leave the VA firewall without a data use agreement. This limitation is consistent with those of other studies based on VA data. However, VA data are freely available to researchers behind the VA firewall, with an approved VA study protocol. For more information, please visit https://www.virec.research.va.gov or contact the VA Information Resource Center (VIReC) at Virec@Va.gov.

## Disclaimer

The authors conducted this study based on personal capacity. The opinions expressed in this article are the authors' own and do not reflect the views of the Department of Veterans Affairs or the United States government.

## Conflicts of interests

BVJ received institutional research support from Exact Sciences, Genentech, and Takeda unrelated to this publication. The remaining authors declare no conflicts of interest that pertain to this work.

Please refer to the accompanying ICMJE disclosure forms for further details.
