# Supplementary material for: Hepatocellular carcinoma risk in metabolic dysfunction-associated steatotic liver disease with increased alcohol intake (MetALD)
Source: JHEP Rep. 2025 Oct 16;8(1):101639. doi: 10.1016/j.jhepr.2025.101639 (PMC12890437; doi:10.1016/j.jhepr.2025.101639)
Supplement: Multimedia component 1 [file mmc1.pdf]

Hepatocellular carcinoma risk in metabolic dysfunction-associated steatotic liver disease with increased alcohol intake (MetALD)

Binu V. John, Dustin Bastaich, Elizabeth Paulus, Seth Spector, Bassam Dahman,

For the Veterans Analysis of Liver Disease (VALID) group of investigators

Table of contents

Table S1……………………………………………………………………………………2

Table S2……………………………………………………………………………………3

Table S3……………………………………………………………………………………4

Table S4……………………………………………………………………………………5

Table S5……………………………………………………………………………………6

Table S6……………………………………………………………………………………7

Table S7……………………………………………………………………………………8

Table S1: Cumulative incidence of HCC at landmark time points by steatosis etiology

	Cumulative Incidence of HCC per 100,000 persons			
**Year**	**MASLD**	**MetALD**	**ALD**	**Control**
**5**	450.71 (428.46, 473.89)	449.89 (407.56, 495.74)	663.79 (581.77, 754.83)	76.03 (61.47, 93.46)
**10**	762.86 (725.86, 801.35)	814.16 (739.99, 894.10)	1176.65 (1033.78, 1334.36)	89.67 (72.61, 110.06)

Note: Unadjusted incidence rates and their confidence intervals were computed using Poisson regression

Table S2: Average number of abdominal imaging exams per patient per year among patients with ALD, MetALD and MASLD.

	Any Exam (US, CT, or MRI)			CT			MRI			Ultrasound		
Year	ALD	MASLD	MetALD	ALD	MASLD	MetALD	ALD	MASLD	MetALD	ALD	MASLD	MetALD
1	2.03	1.77	1.77	0.94	0.90	0.83	0.15	0.15	0.15	0.94	0.72	0.79
2	0.64	0.49	0.48	0.33	0.28	0.26	0.07	0.06	0.06	0.25	0.15	0.17
3	0.57	0.44	0.44	0.29	0.25	0.23	0.06	0.05	0.05	0.23	0.14	0.15
4	0.55	0.42	0.41	0.28	0.24	0.22	0.06	0.05	0.05	0.21	0.13	0.15
5	0.51	0.39	0.40	0.26	0.23	0.21	0.05	0.04	0.05	0.20	0.12	0.14
6	0.49	0.38	0.39	0.26	0.22	0.21	0.06	0.04	0.05	0.18	0.12	0.14
7	0.48	0.37	0.38	0.25	0.21	0.20	0.06	0.04	0.05	0.18	0.11	0.13
8	0.47	0.36	0.37	0.24	0.21	0.20	0.06	0.04	0.05	0.17	0.11	0.13
9	0.44	0.35	0.36	0.23	0.21	0.20	0.05	0.04	0.05	0.16	0.10	0.11
10	0.43	0.33	0.37	0.22	0.19	0.20	0.05	0.04	0.05	0.16	0.10	0.12
11	0.37	0.30	0.34	0.20	0.18	0.18	0.04	0.04	0.05	0.13	0.09	0.11
12	0.28	0.24	0.28	0.15	0.14	0.15	0.03	0.03	0.04	0.10	0.07	0.10

Table S3: Pairwise comparisons of HCC by etiology of steatotic liver disease

	Reference					
	Control		MASLD		MetALD	
	aHR (95% CI) P Value	P Value	aHR (95% CI)	P Value	aHR (95% CI)	P Value
**MASLD**	2.64 (2.46, 2.82) ***<.0001***	***<.0001***				
**MetALD**	2.90 (2.71, 3.10) ***<.0001***	***<.0001***	1.10 (1.05, 1.15)	***<.0001***		
**ALD**	3.11 (2.91, 3.33) ***<.0001***	***<.0001***	1.18 (1.13, 1.24)	***<.0001***	1.07 (1.03, 1.12)	***0.0012***

Note: Method: hazard ratios from multivariable time-updated Fine and Gray competing risk models with death as a competing risk with inverse probability of treatment weighting. Pairwise differences compared with levels of significance at the Bonferroni-adjusted p-value of 0.0083. aHR, adjusted hazard ratio; CI, confidence interval.

Table S4: Adjusted hazard of HCC comparing patients with steatotic liver disease groups, adjusting for cirrhosis defined using ICD 9/10 codes, elevated Fib-4>2.67, or platelet count <150K

Variable	aHR (95% CI)	P Value
**Total Participants**	666,428	
**Number of Events (HCC)**	3,584	
**Etiology (Ref=Control)**		
**MASLD**	5.34 (4.91, 5.81)	***<.0001***
**MetALD**	5.93 (5.45, 6.45)	***<.0001***
**ALD**	6.97 (6.41, 7.59)	***<.0001***
**Age (per 10 years)**	1.26 (1.24, 1.28)	***<.0001***
**Sex (Male vs Female)**	2.52 (2.25, 2.82)	***<.0001***
**Race/Ethnicity (Ref=White)**		
**Black**	0.65 (0.61, 0.69)	***<.0001***
**Hispanic**	1.39 (1.31, 1.47)	***<.0001***
**Other/Missing**	1.09 (1.04, 1.15)	***0.0004***
**Cirrhosis**	6.29 (6.05, 6.54)	***<.0001***
**Diabetes**	1.84 (1.77, 1.91)	***<.0001***
**BMI (Ref=Normal)**		
**Obese**	1.25 (1.19, 1.32)	***<.0001***
**Overweight**	1.18 (1.12, 1.25)	***<.0001***
**Underweight**	0.97 (0.82, 1.15)	0.7072
**Tobacco Use**	0.98 (0.93, 1.02)	0.2504
**ALT**	0.995 (0.995, 0.996)	***<.0001***
**AST**	1.005 (1.005, 1.006)	***<.0001***

Method: multivariable time-updated Fine and Grey competing risk model with death as a competing risk with inverse probability treatment weight, weighted for age, sex, race/ethnicity, and cirrhosis. Age, cirrhosis, diabetes, BMI, tobacco use, ALT and AST levels, and platelet count were updated at the time of cirrhosis in participants who developed cirrhosis at least 90 days after their baseline abdominal imaging examination. BMI, body mass index; ALT, alanine aminotransferase; AST, aspartate aminotransferase; aHR, adjusted hazard ratio; CI, confidence interval; HCC, hepatocellular carcinoma.

Table S5: Adjusted hazard of HCC comparing patients with steatotic liver disease groups, using the updated harmful alcohol use criteria requiring at least 2 of 3 years having an elevated AUDIT-C

Variable	aHR (95% CI)	P Value
**Total Participants**	666,428	
**Number of Events (HCC)**	3,584	
**Etiology (Ref=Control)**		
**MASLD**	2.75 (2.57, 2.94)	***<.0001***
**MetALD**	3.06 (2.86, 3.27)	***<.0001***
**ALD**	3.14 (2.93, 3.36)	***<.0001***
**Age (per 10 years)**	1.47 (1.45, 1.49)	***<.0001***
**Sex (Male vs Female)**	2.54 (2.26, 2.85)	***<.0001***
**Race/Ethnicity (Ref=White)**		
**Black**	0.83 (0.79, 0.88)	***<.0001***
**Hispanic**	1.23 (1.16, 1.30)	***<.0001***
**Other/Missing**	1.08 (1.03, 1.13)	***0.0025***
**Cirrhosis**	12.62 (12.16, 13.10)	***<.0001***
**Diabetes**	1.72 (1.65, 1.78)	***<.0001***
**BMI (Ref=Normal)**		
**Obese**	1.05 (1.01, 1.11)	***0.0303***
**Overweight**	0.99 (0.95, 1.04)	0.7982
**Underweight**	1.15 (1.00, 1.32)	0.0564
**Tobacco Use**	0.94 (0.90, 0.98)	***0.0048***
**ALT**	1.001 (1.000, 1.001)	***0.0362***
**AST**	1.001 (1.001, 1.002)	***<.0001***
**Platelet Count (Ref=>150k)**		
**<50k**	2.40 (2.12, 2.72)	***<.0001***
**50k-150k**	1.75 (1.69, 1.82)	***<.0001***

Method: Method: multivariable time-updated Fine and Grey competing risk model with death as a competing risk with inverse probability treatment weight, weighted for age, sex, race/ethnicity, and cirrhosis. The IPTW was weighted for age, sex, race/ethnicity, and cirrhosis. Age, cirrhosis, diabetes, BMI, tobacco use, ALT and AST levels, and platelet count were updated at the time of cirrhosis in participants who developed cirrhosis at least 90 days after their baseline abdominal imaging examination. BMI, body mass index; ALT, alanine aminotransferase; AST, aspartate aminotransferase; aHR, adjusted hazard ratio; CI, confidence interval; HCC, hepatocellular carcinoma, AUDIT-C; Alcohol Use Disorders Identification Test-Concise.

Table S6: Adjusted hazard of HCC among the subset of patients tested for HBV and HCV

	HBV and HCV Tested	
**Variable**	**aHR (95% CI)**	**P Value**
**Total Participants**	8,363	
**Number of Events (HCC)**	62	
**Etiology (Ref=Control)**		
**MASLD**	REF	
**MetALD**	1.29 (0.92, 1.82)	0.1471
**ALD**	1.76 (1.29, 2.40)	***0.0003***
**Age (per 10 years)**	1.37 (1.25, 1.50)	***<.0001***
**Sex (Male vs Female)**	3.34 (1.55, 7.21)	***0.0021***
**Race/Ethnicity (Ref=White)**		
**Black**	0.87 (0.66, 1.17)	0.3578
**Hispanic**	2.03 (1.42, 2.91)	***0.0001***
**Other/Missing**	1.02 (0.68, 1.54)	0.9213
**Cirrhosis**	5.34 (4.19, 6.80)	***<.0001***
**Diabetes**	1.13 (0.87, 1.47)	0.3708
**BMI (Ref=Normal)**		
**Obese**	1.71 (1.18, 2.47)	***0.0043***
**Overweight**	1.62 (1.14, 2.31)	***0.0072***
**Underweight**	NE	-
**Tobacco Use**	1.17 (0.87, 1.56)	0.2924
**ALT**	1.005 (1.002, 1.008)	***0.0018***
**AST**	0.998 (0.995, 1.001)	0.1243
**Platelet Count (Ref=>150k)**		
**<50k**	NE	-
**50k-150k**	0.96 (0.75, 1.23)	0.7344

Method: multivariable time-updated Fine and Grey competing risk model with death as a competing risk with inverse probability treatment weight. The IPTW was weighted for age, sex, race/ethnicity, and cirrhosis. Age, cirrhosis, diabetes, BMI, tobacco use, ALT and AST levels, and platelet count were updated at the time of cirrhosis in participants who developed cirrhosis at least 90 days after their baseline abdominal imaging examination. BMI, body mass index; ALT, alanine aminotransferase; AST, aspartate aminotransferase; aHR, adjusted hazard ratio; CI, confidence interval; HCC, hepatocellular carcinoma; NE, not estimable

Table S7: Cumulative incidence of HCC at landmark time points by steatosis etiology in female patients

	Cumulative Incidence of HCC per 100,000 persons			
**Year**	**MASLD**	**MetALD**	**ALD**	**Control**
**5**	84.98 (56.96, 124.11)	79.08 (31.00, 181.00)	304.15 (126.66, 651.00)	21.08 (8.34, 48.63)
**10**	201.46 (137.81, 288.14)	179.46 (67.93, 416.57)	893.55 (346.90, 1965.12)	21.08 (8.34, 48.63)

Note: Unadjusted incidence rates and their 95% confidence intervals were computed using Poisson
